# Structure of the human 20S U5 snRNP

**DOI:** 10.1038/s41594-024-01250-5

**Published:** 2024-03-11

**Authors:** Sarah Schneider, Irina Brandina, Daniel Peter, Sonal Lagad, Angelique Fraudeau, Júlia Portell-Montserrat, Jonas Tholen, Jiangfeng Zhao, Wojciech P. Galej

**Affiliations:** 1 European Molecular Biology Laboratory, EMBL Grenoble, Grenoble, France; 2 Present Address: Boehringer Ingelheim RCV GmbH & Co KG, Vienna, Austria; 3 Present Address: Institute of Molecular Biotechnology of the Austrian Academy of Sciences, Vienna BioCenter, Vienna, Austria; 4 Present Address: Research Institute of Molecular Pathology, Vienna BioCenter, Vienna, Austria; 5 Present Address: Department of Structural Biology, Genentech Inc., South San Francisco, CA USA

**Keywords:** Cryoelectron microscopy, Protein-protein interaction networks, RNA splicing

## Abstract

The 20S U5 small nuclear ribonucleoprotein particle (snRNP) is a 17-subunit RNA–protein complex and a precursor of the U4/U6.U5 tri-snRNP, the major building block of the precatalytic spliceosome. CD2BP2 is a hallmark protein of the 20S U5 snRNP, absent from the mature tri-snRNP. Here we report a high-resolution cryogenic electron microscopy structure of the 20S U5 snRNP, shedding light on the mutually exclusive interfaces utilized during tri-snRNP assembly and the role of the CD2BP2 in facilitating this process.

## Abstract (web summary)

Here the authors report the structure of the human 20S U5 snRNP, providing new insights into the assembly of the spliceosome building blocks.

## Main

In eukaryotes, the removal of noncoding introns from pre-mRNAs is catalyzed by the large and dynamic spliceosome complex^1^. The spliceosome assembles de novo on each intron from small nuclear ribonucleoprotein particles (snRNPs) and numerous protein factors. The 39-subunit U4/U6.U5 tri-snRNP is the largest preassembled building block of the spliceosome, which joins the splicing pathway at the precatalytic (pre-B) stage and delivers two components of the RNA catalytic core, the U5 and U6 snRNAs, in their inactive configurations requiring further remodeling^2,3^. Despite recent progress in the mechanistic understanding of spliceosome assembly, the biogenesis and recycling of its building blocks remain elusive.

Several factors associate with the tri-snRNP components but are absent in mature particles; hence, they are believed to play roles in tri-snRNP biogenesis and/or recycling. These include the U5 snRNP binding proteins: AAR2 (refs. ^4,5^), CD2BP2 (U5-52K; *Saccharomyces cerevisiae* Lin1)^6,7^, TSSC4 (refs. ^8,9^) and ZNHIT2 (refs. ^10–12^), as well as the U4/U6 annealing factor SART3 (*S.cerevisiae* Prp24)^13–15^. Mechanistically, the exact roles and the interplay of these assembly factors remain poorly understood.

The 20S U5 snRNP isolated from HeLa cells contains at least 16 subunits, including its hallmark protein CD2BP2 (refs. ^16,17^), which also plays a role in the binding of the CD2 receptor^18^. Conditional knockout (KO) of CD2BP2 in mice leads to growth defects and premature death during embryonic development^19^. In its role as a splicing factor, CD2BP2 binds to DIM1 (U5-15K) with its GYF domain, forming a protein–protein interface that differs from the canonical binding mode to sequence motifs in the CD2 antigens^16,18^. Lin1, the yeast homolog of CD2BP2, was reported to bind PRP8, suggesting a possible mode of its recruitment to the U5 snRNP complex^20^.

Although 20S U5 snRNP was first isolated several decades ago^17^, its molecular structure and the function of CD2BP2 remain unknown. In this Brief Communication, we investigate how CD2BP2 interacts with other components of the U5 snRNP and how it facilitates tri-snRNP formation.

**Fig. 1:**
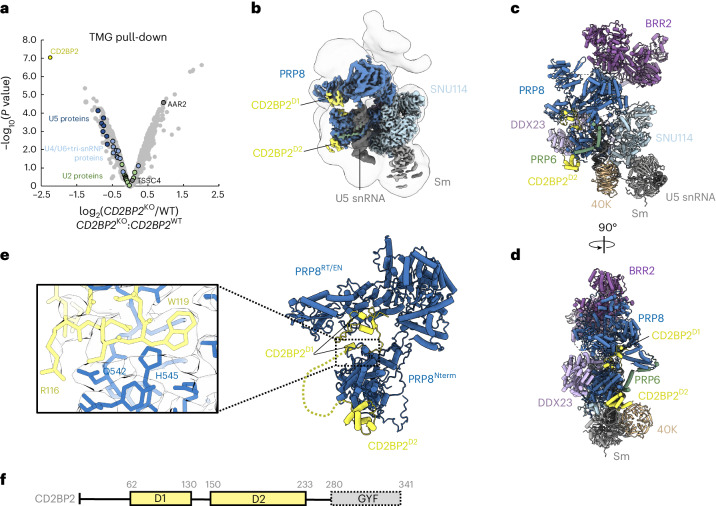
Cryo-EM structure of the 20S U5 snRNP. **a**, Quantitative mass spectrometry analysis of snRNPs isolated via TMG agarose from WT or CD2BP2-KO HEK293T cells. A moderated two-sided *t*-test was applied for statistical analysis. **b**, Experimental cryo-EM map of the 20S U5 snRNP colored by the subunit identity fitted into the low-pass filtered map at the lower contour level. **c**, Atomic model of the 20S U5 snRNP shown in the same orientation as in **b**. **d**, Orthogonal view of the atomic model. **e**, Zoomed-in view of the PRP8–CD2BP2 interaction highlighting the extended hook-like domain. **f**, Domain architecture of CD2BP2. Source data

**Extended Data Fig. 1:**
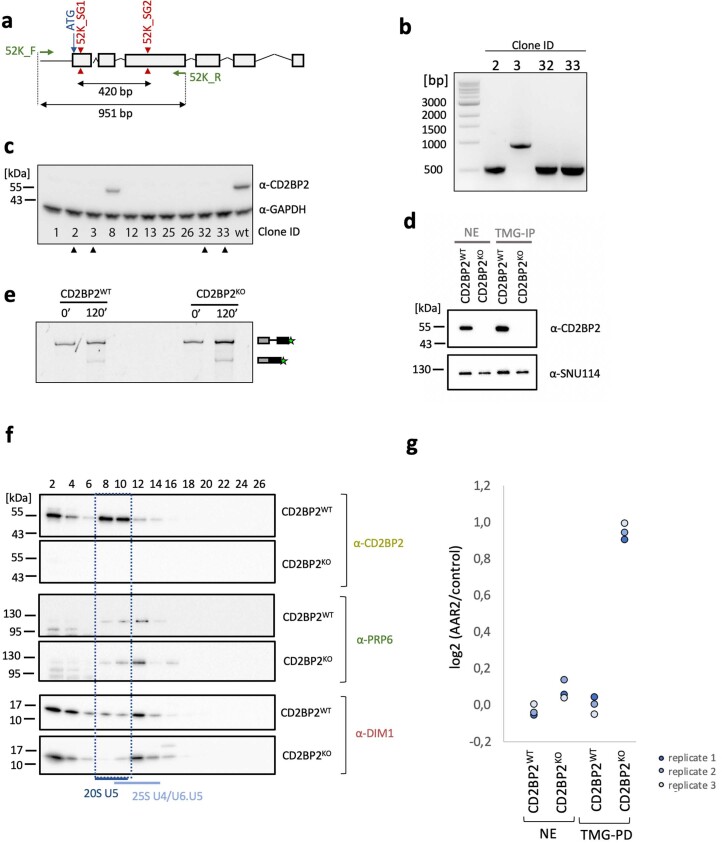
CD2BP2 knock out and its impact on pre-mRNA splicing and snRNPs assembly. **a**, experimental design used in CRISPR/Cas9-mediated CD2BP2^KO^ generation; **b**, genotyping of the CD2BP2^KO^ clones using PCR as indicated in panel a; **c**, Western blot analysis of the knockout cell lines with anti-CD2BP2 antibodies and GAPDH used as a loading control; **d**, Western blot of the NE and TMG pull-down fractions used in Fig. 1 a probed for the presence of CD2BP2 and SNU114; **e**, *In vitro* splicing assay of the AdML-M3 pre-mRNA substrate in the nuclear extract prepared from WT or CD2BP2^KO^ cell lines; **f**, Western blot of the glycerol gradient fractions probed with U5 snRNP-specific antibodies, showing depletion of DIM1 from 20 S U5 snRNP in CD2BP2 KO condition; **g**, Quantitative mass spectrometry measurement of the AAR2 abundance in the input nuclear extracts (NE) and TMG pull-down fractions (TMG-PD) from WT and CD2BP2 knock-out cell lines. Experiments in b-e were performed in single biological replicates, while d-g were done in triplicates with consistent outcomes. Source data

First, we analyzed the steady-state composition of the spliceosomal snRNPs in the absence of CD2BP2. We purified snRNP using anti-2,2,7-trimethylguanosine (TMG) antibody-coupled resin from nuclear extracts (NE) prepared either from wild-type (WT) HEK293T cells or a homozygous CRISPR–Cas9 CD2BP2 KO cell line (*CD2BP2*^KO^; Extended Data Fig. 1a–c). The composition of both samples was compared by quantitative mass spectrometry (Fig. 1a). As expected, we observe a clear depletion of the CD2BP2 in the KO sample as well as a subtle, but consistent, underrepresentation of nearly all U5 snRNP subunits in the KO condition. U4/U6 and tri-snRNP specific components are also affected, yet, to a lesser extent, while the U2 snRNP proteins remained virtually unchanged, as their assembly into snRNPs is not expected to depend on CD2BP2. As such, these results point to a subtle defect in the U5 and U4/U6.U5 tri-snRNP assembly in the absence of CD2BP2. Yet, we could not observe a noteworthy impact on the cell viability and in vitro splicing efficiency under the conditions tested (Extended Data Fig. 1e). Interestingly, another U5 snRNP assembly factor, AAR2, is significantly enriched in snRNPs isolated from the *CD2BP2*^KO^ cells. This could be due to the upregulation of the AAR2 in the absence of CD2BP2 or due to a failure in the CD2BP2-dependent conversion of AAR2-containing U5 snRNP into 20S U5 snRNP during the biogenesis. The latter seems more probable, as the expression levels of AAR2 in NE of WT and KO cell lines are largely unchanged (Extended Data Fig. 1g). This data provide evidence that CD2BP2 is indeed involved in the U5 snRNP assembly and establish a functional link to another U5 snRNP assembly factor AAR2.

**Fig. 2:**
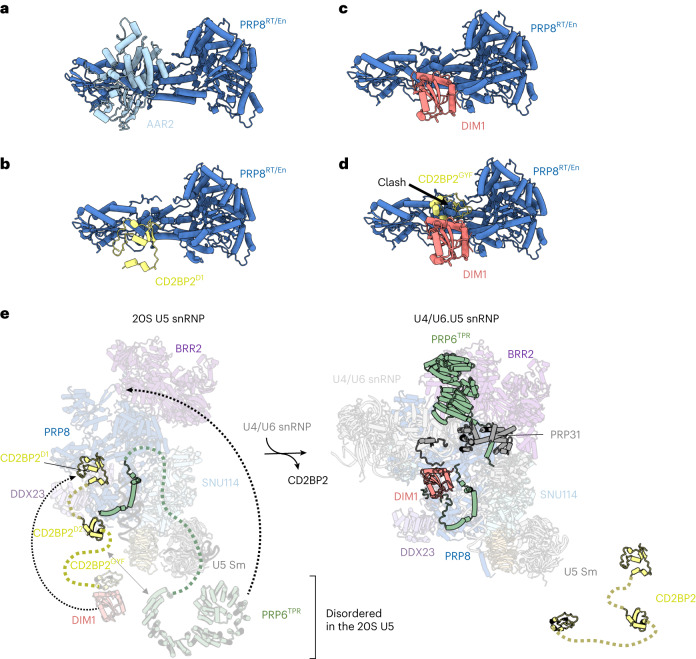
Mutually exclusive interactions of assembly factors with PRP8 during tri-snRNP formation. **a**, AAR2 binding mode to PRP8^RT/EN^ domain^23^. **b**, CD2BP2^D1^ occupies an overlapping surface of PRP8 in 20S U5 snRNP. **c**, DIM1 binding site in tri-snRNP^21^ is mutually exclusive with CD2BP2^D1^. **d**, PRP8 binding surface on DIM1 is occupied by the CD2BP2^GYF^ domain in the CD2BP2–DIM1 binary complex^16^ and clashes with PRP8 when superimposed on DIM1 in tri-snRNP. **e**, A structural model of the tri-snRNP assembly. Left: an atomic model of the 20S U5 snRNP including the disordered DIM1, CD2BP2^GYF^ and PRP6^TPR^ domains. Right: an atomic model of the fully assembled tri-snRNP^21^. Recruitment of the U4/U6 di-snRNP to 20S U5 snRNP triggers the relocation of PRP6, allowing DIM1 to compete with CD2BP2 and displace it from its binding site on PRP8.

**Extended Data Fig. 2:**
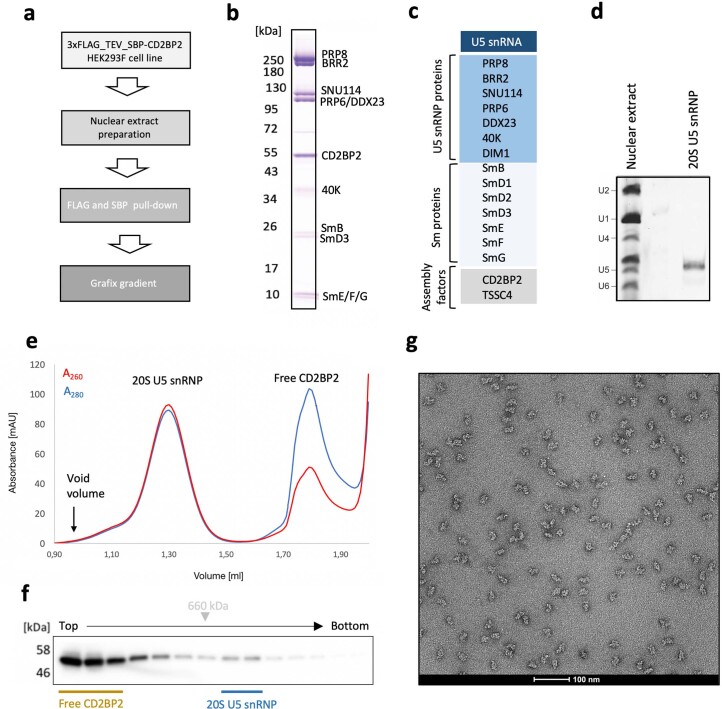
Purification of the 20S U5 snRNP from HEK293F cells. **a**, experimental workflow used in sample preparation; **b**, SDS-PAGE analysis of the SBP eluates used subsequently for the Grafix gradient; **c**, a list of proteins present in the 20S U5 snRNP preparation; **d**, UREA-PAGE analysis of the RNAs extracted from SBP eluate of the CD2BP2-purified sample or NE used as a control. RNAs were stained with SYBR Gold and detected using Bio-Rad Chemidoc; **e**, 20S U5 snRNP sample analysed on the Superose 6 3.2/300 size exclusion chromatography column; **f**, the same sample analysed on a non-cross-linking 10–30% glycerol gradient and detected by Western blot with anti-FLAG antibodies. The migration of the thyroglobulin size marker is indicated with the grey arrow; **g**, a typical micrograph of negatively stained Grafix fractions used subsequently for cryo-EM analysis. Experiments in panels b,d,g and f, were performed in triplicates with consistent outcomes. Source data

**Extended Data Fig. 3:**
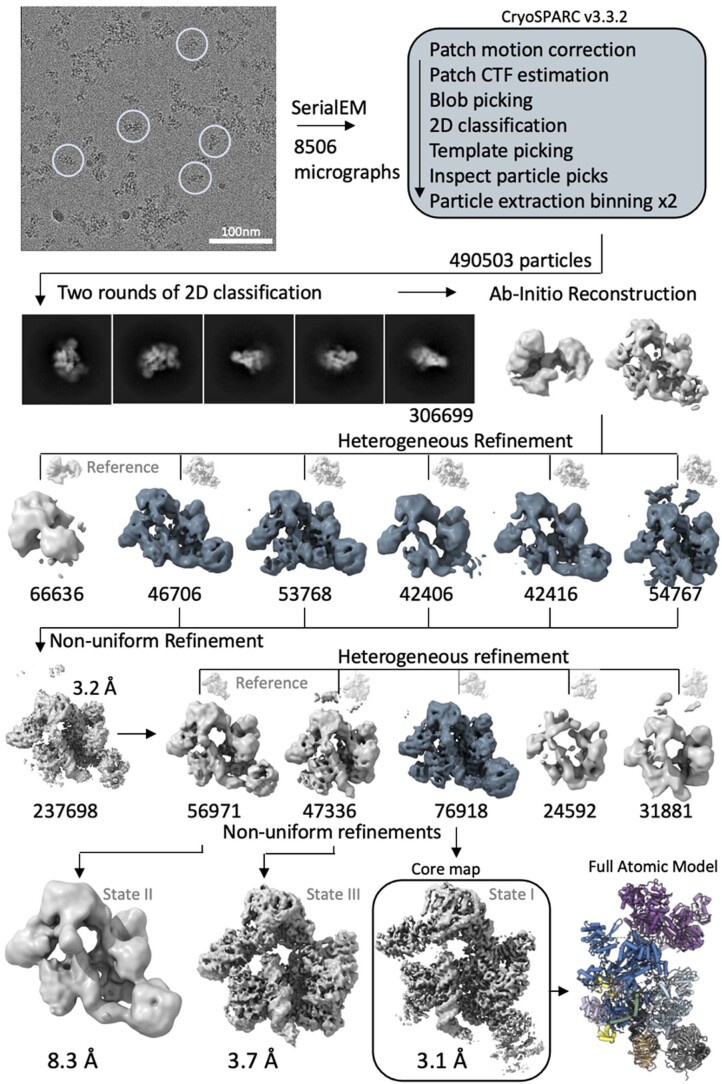
Cryo-EM data processing chart. Processing chart for the 20S U5 snRNP. The dataset contained compositionally heterogeneous snRNP, which were separated by heterogeneous refinement. This was then followed by non-uniform refinement. References are depicted in grey. Numbers indicate particle numbers unless otherwise stated. Extended Data Fig. 6 contains a more detailed comparison of the final three reconstructions.

**Extended Data Fig. 4:**
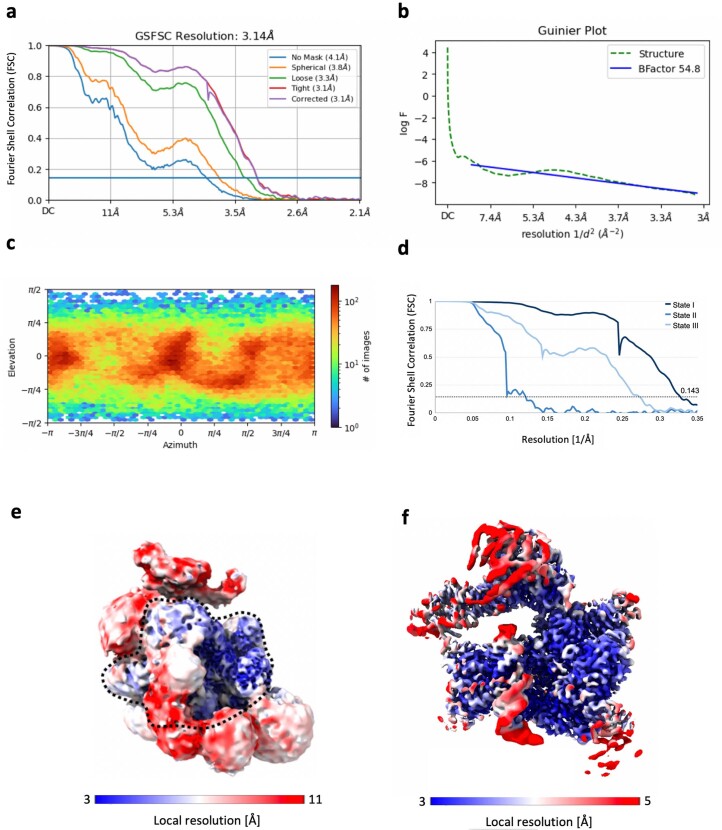
Global and local resolution analysis of the cryo-EM reconstructions obtained in this study. Graphs based on the 3.1 Å, determined with cryoSPARC v3.3.2 unless otherwise indicated. **a**, Fourier Shell Correlation (FSC) of the final 3.1 Å cryo-EM map with different masks; **b**, Guinier plot used to determine the b-factor of 54.8 Å^2^; **c**, Angular distribution shows no major preferential orientation; **d**, FSC curves of the final three reconstructions; **e**, Local resolution plotted on the isosurface of the locally filtered state I map at a low contour level. The core of the particle shown in panel **f**, is indicated with a dotted line.

**Extended Data Fig. 5:**
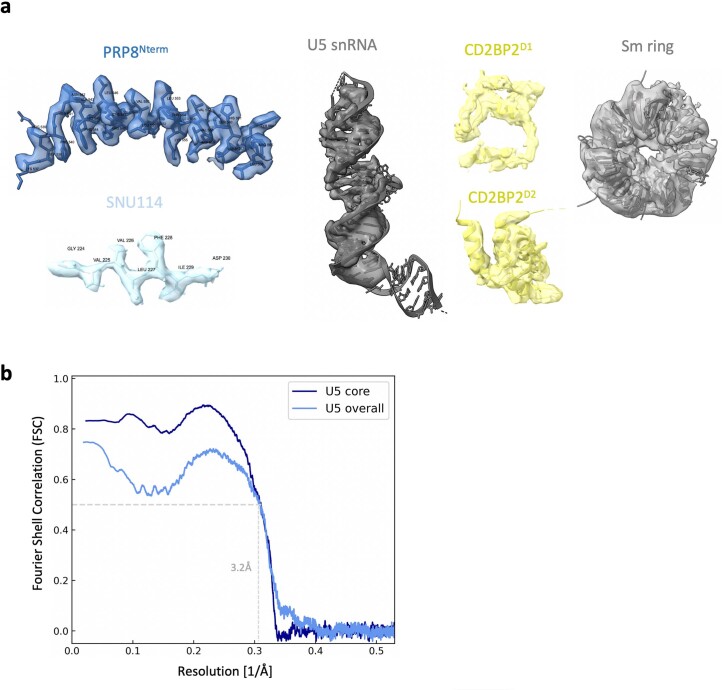
Examples of the atomic model fitting into the cryo-EM density of the 20S U5 snRNP. **a**, Representative examples of PRP8 and SNU114, U5 RNA and CD2BP2 areas resolved at high-resolution. The Sm ring is an example of a low-resolution fitting. **b**, Fourier Shell Correlation (FSC) of the model vs map shows good agreement up to 3.2 Å (FSC = 0.5 cut-off).

**Extended Data Fig. 6:**
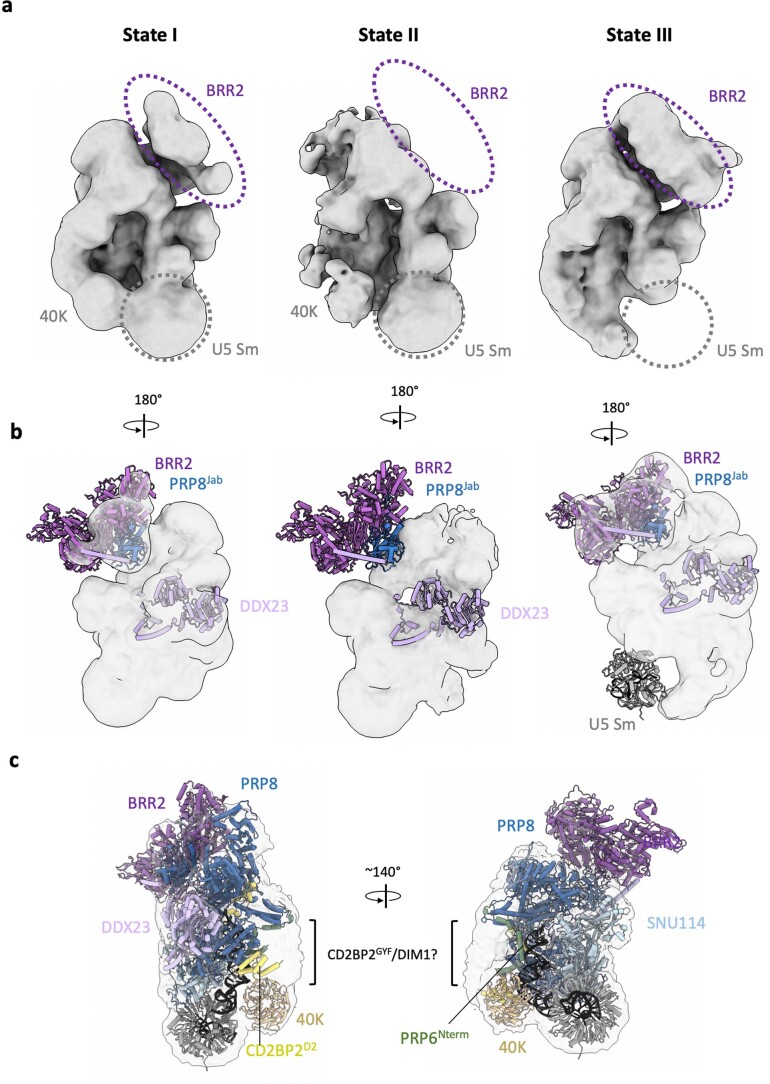
Three major compositional states are present in the reconstruction of the 20S U5 snRNP. **a**, Low-pass filtered reconstructions of the three states present in the reconstruction highlighting the key differences between them; **b**, Atomic models of the highlighted components fitted into the corresponding maps. State I represents the fully assembled complex referred to as the 20S U5 snRNP; state II has overall very similar architecture, but no clear density for BRR2 and DDX23 was observed; state III misses Sm ring and 40K and most likely represents broken particles. A comparison of state I and state II shows that BRR2 and DDX23 stabilise each other, as their presence is largely correlated. It has been previously shown that DDX23 binding to BRR2 is mutually exclusive with TSSC4 (ref. ^9^). Therefore, the absence of DDX23/BRR2 in one of the classes could, in principle, represent a state where TSSC4 is bound to BRR2 and prevents its DDX23-mediated stable docking to the body of the complex. However, we could not see density for TSSC4 in any of these reconstructions even though putative TSSC4-PRP8 interface could be predicted with AlphaFold2; **c**, Unassigned density located near 40K, CD2BP2 and PRP8 most likely represents DIM/CD2BP2^GYF^ domain.

**Extended Data Fig. 7:**
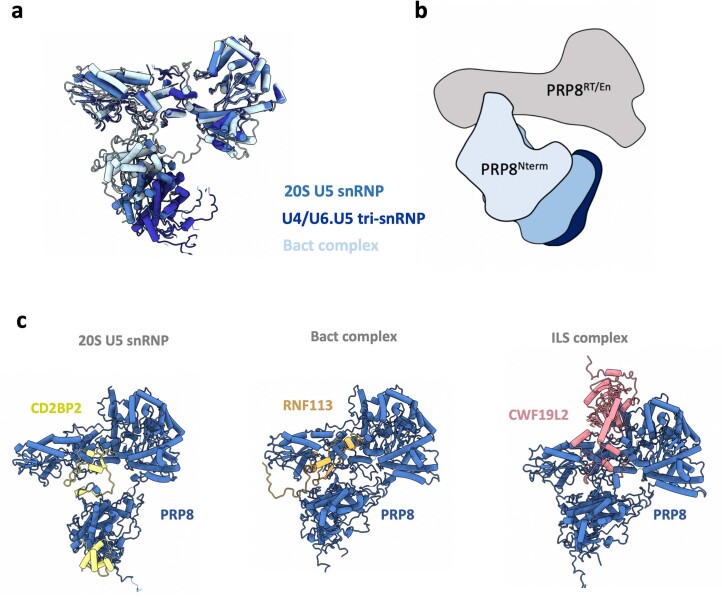
PRP8 conformation in the 20S U5 snRNP. **a**, superposition of PRP8 from 20S U5 snRNP structure (this work), U4/U6.U5 tri-snRNP^21^ (PDB:6QW6) and Bact spliceosome^24^ (PDB:6FF4) showing the movement of the PRP8^Nterm^ with respect to PRP8^RT/EN^ in different splicing complexes. The position of this domain in 20S U5 snRNP is likely stabilised by CD2BP2^D1^; **b**, a cartoon representation highlighting the movement described in panel a. **c**, CD2BP2 binding surface on PRP8^RT/EN^ domain is occupied by different factors in other splicing complexes: RNF113 in Bact spliceosome^24^ and CW19L2 in post-splicing ILS complex^25^.

Next, to gain insights into the structure of the 20S U5 snRNP, we engineered HEK293F cells to express a 3xFLAG_TEV_SBP-tagged CD2BP2 and used it to purify a 17-subunit complex containing the U5 snRNA, seven Sm core proteins and nine other factors (Extended Data Table 1 and Extended Data Fig. 2). The composition of the complex is in good agreement with previous reports^7,17^ and, interestingly, includes an additional assembly factor, TSCC4 (not resolved in the structure)^8,9^. We determined a cryogenic electron microscopy (cryo-EM) structure of the CD2BP2-bound 20S U5 snRNP complex at the 3.1 Å resolution (Fig. 1, Table 1, Methods and Extended Data Figs. 3–5). The architecture of the 20S U5 snRNP closely resembles that of the U5 snRNP captured as a part of the tri-snRNP^2,21^ and in low-resolution U5 snRNP studies^22^. At least three major states are present in our 20S U5 snRNP reconstruction (Extended Data Fig. 6). State I contains most of the components and is referred to as the 20S U5 snRNP hereafter. State II is missing two helicases, BRR2 and DDX23, and may represent an earlier stage of the U5 snRNP assembly (Extended Data Fig. 6). State III contains particles missing the Sm ring, most probably damaged during the vitrification process. In all reconstructions, PRP8 provides the scaffold for the entire complex and interacts with multiple other subunits (Fig. 1). PRP6 is present in the sample, but only its N-terminal helices are visible, and the tetratricopeptide (TPR) repeat remains disordered. We observed two well-defined densities located near PRP8^RT/En^ and PRP8^Nterm^ domains, which were assigned to CD2BP2 domains D1 (62-130) and D2 (150-233), respectively (Fig. 1). The CD2BP2^GYF^ domain (280-341) and its binding partner DIM1 are not visible in our structure. A hook-shaped extension of the CD2BP2^D1^ bridges the PRP8^RT/En^ and PRP8^Nterm^ domains and probably stabilizes their relative orientation, which differs from the one observed in the tri-snRNP (Fig. 1 and Extended Data Fig. 7a,b). CD2BP2^D1^ occupies the surface of PRP8 that accommodates several different factors during the splicing cycle, including AAR2 in the U5 snRNP precursor^5,23^, DIM1 in tri-snRNP and the precatalytic spliceosome^2,21^, as well as RNF113 in Bact^24^ and CWF19L2 in the postsplicing ILS complexes^25^ (Fig. 2 and Extended Data Fig. 7c).

**Table 1:** Cryo-EM data collection, refinement and validation statistics

	20S U5 snRNP (complete) (EMD-19041) , (PDB 8RC0)	20S U5 snRNP (core) (EMD-18267) , (PDB 8Q91)
**Data collection and processing**		
Magnification	130,000	
Voltage (kV)	300	
Electron exposure (e^−^ Å^−^^2^)	40.5	
Exposure rate (e^−^ per pixel per second)	4.57	
Defocus range (μm)	1.5–3.5	
Pixel size (Å)	1.045	
Symmetry imposed	C1	
Movies collected (no.)	8506	
Initial particle images (no.)	490,503	
Final particle images (no.)	76,918	
Map resolution (Å)	3.1	
FSC threshold	0.143	
Map resolution range (Å)	2.7–15	
**Refinement**		
Initial model used (PDB code)	6qw6	6qw6
Model resolution (Å)	3.2	3.2
FSC threshold	0.5	0.5
Model resolution range (Å)	2.7–15	2.7–5
Map sharpening B-factor (Å^2^)	−55	−55
Model composition		
Nonhydrogen atoms	42,341	28,634
Protein residues	6,394	3,316
Ligands	1	1
B-factors (Å^2^)		
Protein	157.6	54.4
RNA	243.6	87.2
Root mean square deviations		
Bond lengths (Å)	0.007	0.005
Bond angles (°)	0.796	0.870
Validation		
MolProbity score	2.16	2.21
Clashscore	11.6	14.3
Poor rotamers (%)	3.1	2.2
Ramachandran plot		
Favored (%)	96.68	95.90
Allowed (%)	3.26	4.04
Disallowed (%)	0.06	0.06

**Extended Data Fig. 8:**
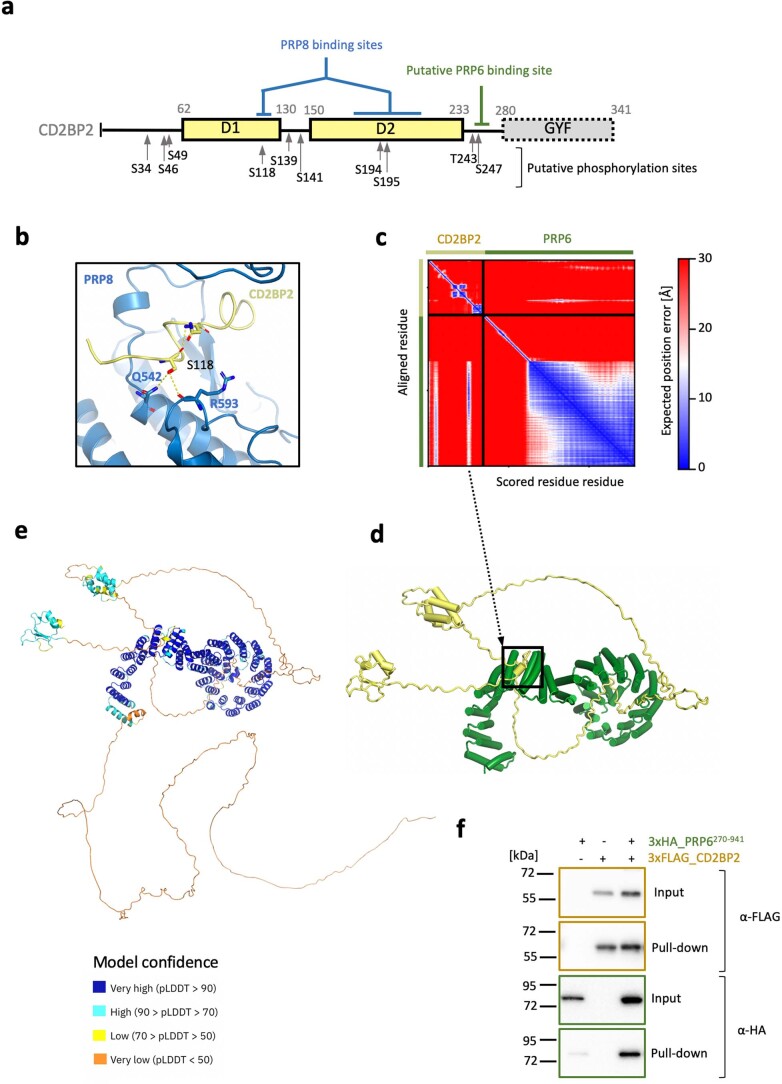
Putative CD2BP2-PRP6 interface and phosphorylation sites in CD2BP. **a**, putative phosphorylation sites in CD2BP2 detected in high-throughput experiments^53,54^ mapped on the primary sequence representation; **b**, Interaction network of CD2BP2 Serine 118, a putative phosphorylation site that is well resolved in the structure; **c** and **d**, a cartoon representation and the PAE plot of the AlphaFold2 model of the CD2BP2-PRP6 binary complex; **e**, AlphaFold2 model of the full-length PRP6-CD2BP2 complex coloured based on pLDDT score (prediction confidence metric); **f**, Western blot analysis of the FLAG-tag pull-down experiment from HEK293T cells co-expressing 3xFLAG-CD2BP2 and 3xHA-PRP6^TPR^, confirming direct interaction between the two proteins. Experiment in panel f was performed in a single biological replicate. Source data

**Extended Data Fig. 9:**
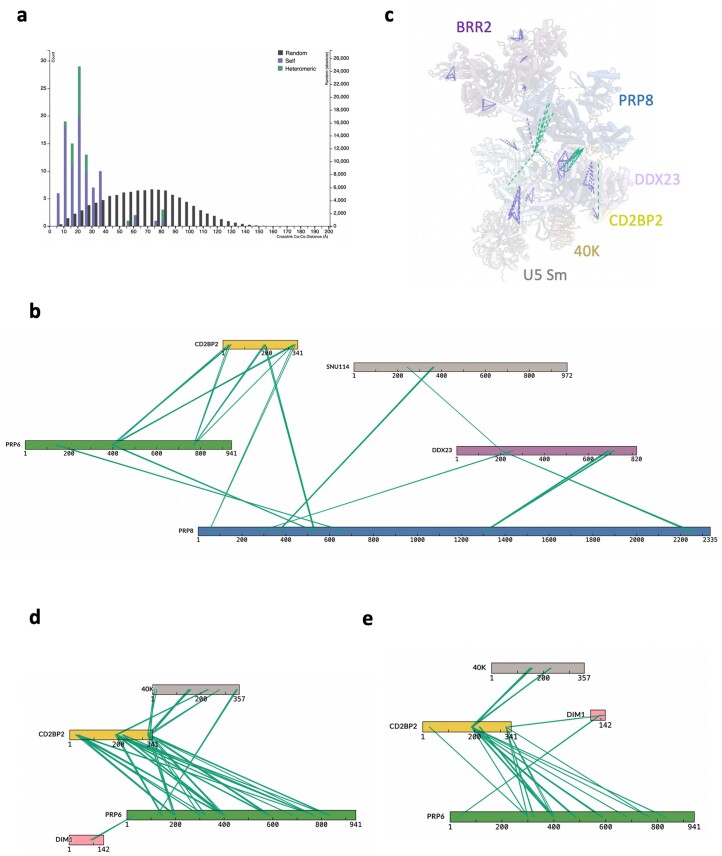
BS3 cross-linking and mass spectrometry analysis of the 20S U5 snRNP. **a**, distribution of the Cα-Cα distances mapped on the core U5 snRNP structure. The majority of the inter- and intra-molecular cross-links fall within 35 Å distance, consistent with the chemical nature of the cross-linker; **b**, visualisation of the high-confidence cross-links (Match score > 180) with respect to their position in their target proteins; **c**, mapping of the high-confidence cross-links onto the structure of the 20S U5 snRNP; **d** and **e**, unfiltered cross-links from two independent experiments (0.25 mM and 1 mM BS3) mapped on the sequences of CD2BP2, DIM1, PRP6 and 40K. These contacts support the model, wherein C-terminus of CD2BP2 and DIM1 are located near 40K. PRP6 TPR repeats appear to form multiple cross-links with CD2BP2, consistent with the AF2 model and biochemical data (Extended Data Fig. 8). XL-MS data was analysed and visualized in the xiView^52^. Source data

Interestingly, the CD2BP2^GYF^ domain delivers DIM1 to the U5 snRNP, which then competes with CD2BP2^D1^ for the same binding site on PRP8. The interface of DIM1 that contacts PRP8 in tri-snRNP is most probably occupied by the CD2BP2^GYF^ domain, as shown in the crystal structure^16^. Therefore, CD2BP2 constitutes a two-layered buffer blocking the DIM1–PRP8 interaction by simultaneously binding to the interfaces on both PRP8 and DIM1 (Fig. 2). Based on our structural data, we believe that at least two functions of CD2BP2 should be considered. First, it facilitates the recruitment of PRP6 and DIM1, both of which are critical for the tri-snRNP formation. PRP6, together with PRP8, forms an interface that is necessary for PRP31 (and U4/U6 di-snRNP) anchoring, which is then further stabilized by the PRP6^TPR^ domain, forming a bridge between U4/U6 and U5 snRNPs (Fig. 2). PRP6 and CD2BP2 interact directly^7^ (Extended Data Figs. 8 and 9). Therefore, CD2BP2-mediated prerecruitment of PRP6 to the U5 snRNP would probably enhance the efficiency of the tri-snRNP formation. Second, CD2BP2 acts as a placeholder preventing PRP8^RT/En^ association with its numerous binding partners in a wrong spatiotemporal context. A similar mechanism is utilized by some factors involved in ribosome biogenesis^26^. Both of the above functions could serve to ensure the correct order of events during the formation of complex intersubunit interfaces within tri-snRNP.

The remaining question is how CD2BP2 displacement is regulated. Our data shows that DIM1 competes with CD2BP2 for the same binding site on PRP8. We could not locate DIM1 in our map, but we observed some additional, low-resolution density near U5 snRNA, which most probably belongs to DIM1/CD2BP2^GYF^ (Extended Data Fig. 6 and 9). Our cross-linking mass spectrometry (XL-MS) data detect DIM1–CD2BP2^GYF^ and 40K–CD2BP2^GYF^ interactions consistent with this putative location (Extended Data Fig. 9). Therefore, it is possible that DIM1 remains constrained in this position and cannot engage in the competition with CD2BP2^D1^. Recruitment of the U4/U6 di-snRNP would trigger a large-scale movement of PRP6 (Fig. 2). Since PRP6 and CD2BP2 interact with one another (Extended Data Figs. 8 and 9), such movement of PRP6 could exert a force on CD2BP2, displacing it from PRP8 and liberating DIM1, allowing it to adopt its final location.

It has been previously shown that CD2BP2 undergoes phosphorylation, which in principle, could also regulate its displacement^27,28^. One of the putative phosphorylation sites lies at the interface between CD2BP2^D1^ and PRP8^Nterm^ (Extended Data Fig. 8) and could potentially modulate their affinity. However, phosphorylation of CD2BP2 does not appear necessary for the in vitro reconstitution of the tri-snRNP, and its function remains unclear^27^.

Although our data indicate that CD2BP2 is required for the tri-snRNP formation and acts downstream of AAR2, we cannot discriminate whether its role concerns predominantly the initial biogenesis of the U5 snRNP or its potential recycling from postsplicing complexes. As such, more work is required to shed light on the interplay between these two factors and their roles in respective pathways.

## Methods

### CD2BP2 KO cell line generation

Two guide RNAs were designed to delete 420 bp of the genomic locus near the translation start site of the CD2BP2 gene and cloned into the PX458 vector (pSpCas9(BB)-2A-GFP; a gift from Feng Zhang; Addgene plasmid no. 48138) using pairs of annealed oligonucleotides as follows:

SG1_FW:CACCGaaagtgaccttccaaggcgt+SG1_REV:aaacacgccttggaaggtcactttC; SG2_FW:CACCGACACTCTTTGGATAGCGATG+SG2_REV:aaacCATCGCTATCCAAAGAGTGTC.

HEK293T cells (ATCC CRL-3216) were seeded in a 6-well plate at a density of 0.3 × 10^6^ cells per well and incubated for 24 h in Dulbecco’s modified Eagle medium (Gibco) supplemented with 5% fetal bovine serum and penicillin/streptomycin (Thermo Fisher Scientific). Then, 1 µg of each guide RNA-containing PX458 plasmid was transfected into the cells using Lipofectamine 2000 (Thermo Fisher Scientific), following the manufacturer recommendations. After 5 days of growth, cells were trypsinized with trypsin-EDTA 0.05% (Thermo Fisher Scientific), and single cells showing green fluorescent protein (GFP) signal were sorted into 96-well plates using a BD FACSAria IIu (BD Biosciences) sorter. Clonal cell lines were expanded over the period of 2 weeks and analyzed for the presence of the desired deletion using polymerase chain reaction (PCR; 52K_FW: GATCCAGAGGGTCCGCTCC; 52K_REV: CCTTCCTCCATCTCCTCCTGC) and western blotting with anti-CD2BP2 antibodies (Thermo Fisher Scientific; PA5-59603; RRID:AB_2639539).

### TMG agarose immunoaffinity chromatography and quantitative mass spectrometry

NEs from HEK293T WT and *CD2BP2*^KO^ were prepared following the original Dignam protocol^29^. TMG agarose beads (TMG mouse antibodies, K121, agarose conjugate, Merck NA02A) were preblocked by incubation with 0.1% BSA in phosphate-buffered saline (PBS) buffer for 1 h at 8 °C, then washed with two bead volumes (CV) of the immunoprecipitation (IP) buffer (20 mM Tris-HCl pH 7.9; 150 mM KCl) supplemented with protease inhibitor cocktail (Roche cOmplete). TMG-beads were added to NEs to the final volume of 10% and incubated ON at 8 °C, with shaking. TMG-beads were collected in Mini Bio-Spin chromatography columns, Bio-Rad, by centrifugation for 30 s at 2,500*g* at 8 °C. After two wash steps with the IP buffer, beads were eluted by boiling for 10 min with the buffer containing 50 mM Tris-HCl pH 7.9, 150 mM KCl and 0.5% sodium dodecyl sulfate (SDS). The eluates from the experiment performed in triplicates were analyzed by a TMT-plex quantitative mass spectrometry as previously described^30^.

### Western Blotting

For the TMG pull-down eluates and NEs, equal amounts of total protein or fractions after glycerol gradient were separated by sodium dodecyl sulfate polyacrylamide gel electrophoresis using WedgeWell 4–20% Tris-glycine system, Invitrogen. The transfer to the polyvinylidene difluoride (PVDF) membrane was done in the Trans-Blot Turbo system, Bio-Rad, using a Turbo-transfer buffer. The following primary rabbit polyclonal antibodies were used: CD2BP2 (Sigma, HPA061309), DIM1 (Proteintech, 27646-1-AP), PRP6 (Invitrogen, PA5-61428) and SNU114/EFTUD2 (Invitrogen, PA5-96559). The secondary antibody was goat anti-rabbit IgG HRP-conjugate (Abcam, ab205718). Mouse monoclonal conjugated antibodies were anti-FLAG M2-peroxidase (Sigma, A8592), anti-GAPDH (Invitrogen MA515738HRP) and anti-HA-Tag F-7 HRP-conjugate (SantaCruz, sc-7392). The blots were visualized using Pierce ECL Western Blotting Substrate, Thermo Fisher Scientific, and documented on the ChemiDoc MP imaging system and ImageLab, Bio-Rad.

For the PRP6-CD2BP2 pull-down experiment, HEK293T cells were seeded into 6-well plates 24 h before transfection at a density of 500,000 cells per well in 1.5 ml Dulbecco’s modified Eagle medium medium with 10% fetal bovine serum. Plasmids containing 3xHA_PRP6^270-941^ and 3xFLAG_CD2BP2, both under CMV promoters, were mixed 1:1 and a total of 1 μg of DNA was diluted into in 50 μl of opti-MEM and mixed with 3 μg of polyethylenimine (PEI) MAX 40K in 50 μl of opti-MEM and incubated at room temperature for 10 min. Transfection solutions were added drop by drop to each well. The cells were collected by centrifugation 48 h after transfection, lysed in 400 μl of lysis buffer (150 mM KCl, 20 mM K-HEPES pH 7.8 and 0.1% Triton X-100) and sonicated for 10 s at 30% amplitude. The lysates were cleared by centrifugation in a table-top centrifuge at 20,000*g* at 4 °C for 30 min. The supernatant was incubated for 2 h with 5% (v/v) of FLAG-agarose to capture the bait protein. Affinity resin was washed three times with ten resin volumes of buffer 3 (150 mM KCl and 20 mM K-HEPES pH 7.8) and subsequently resuspended in SDS sample buffer and heated up to 95 °C for 5 min to release bound proteins. Input and elution fractions were analyzed by western blotting.

A PVDF membrane (Merck) was activated for 5 min in 100% EtOH and incubated for 5 min in the transfer buffer (1× Tris-glycine, 20% EtOH). A wet transfer was performed for 60–90 min at 30 V in an Invitrogen XCell II Blot Module. The membrane was blocked with 5% milk in PBS supplemented with 0.2% Tween 20 (PBST) for 1 h at room temperature. Primary antibodies were added in the following dilutions: anti-HA 1:5,000 ((HA-7) HRP ab49969, Abcam); anti-FLAG 1:5,000 (HRP sigma A8592-.2MG). The membrane was washed three times for 5 min with 20 ml of PBST, and chemiluminescence was detected with an HRP substrate kit (Pierce ECL Western Blotting Substrate) in a ChemiDoc imager (Bio-Rad).

### In vitro splicing assay

AdML-M3 pre-mRNA substrate was obtained by run-off in vitro T7-transcription^31^, capped by VCE, NEB and labeled with fluorescein-5-thiosemicarbazyde at the 3′ end as previously described^32^. NEs prepared from WT or *CD2BP2*^KO^ cells were used. The typical reaction contained 30 mM KCl, 3 mM MgCl_2_, 2 mM ATP, 20 mM creatine phosphate, 20 nM RNA AdML_M3 RNA substrate and 40% NE. Splicing reactions were assembled in 20 µl volume and incubated for 2 h at 30 °C. RNA was then isolated by phenol/chloroform extraction and ethanol precipitation and analyzed by denaturing 6% polyacrylamide gel electrophoresis in 7 M urea. Fluorescence of the RNA substrate and splicing product was visualized on ChemiDoc MP.

### 3xFLAG_TEV_SBP_CD2BP2 cell line generation

Open Reading Frame of CD2BP2 was cloned into a modified pFLAG_CMV10 vector containing an N-terminal 3xFLAG_TEV_SBP affinity tag. FreeStyle 293-F cells were transfected with this plasmid, and a stable, polyclonal cell line was derived through G418 antibiotic selection. Expression of the target protein was confirmed by western blot analysis.

### Purification of the 20S U5 snRNP for cryo-EM analysis

Suspension culture of FreeStyle 293-F cells was grown in the FreeStyle medium (Thermo Fisher Scientific) to the density of ~2 × 10^6^ cells ml^−1^ in an orbital shaker (Infors) at 37 °C, 8% CO_2_ and 90 rpm. The cell culture was collected by centrifugation, and NE was prepared following the original Dignam protocol^29^. After the final dialysis step, samples were aliquoted and flash-frozen in liquid nitrogen. For each preparation, an aliquot of NE was thawed on ice, and the salt concentration was adjusted to the final 500 mM KCl. EZview Red anti-FLAG M2 Affinity Gel (Sigma) was added to 10% (v/v) of the reaction volume and incubated overnight at 8 °C with shaking. The resin was washed three times with 10 column volumes (CV) of the wash buffer 1 (20 mM K-HEPES pH 7.9, 500 mM KCl, 2 mM MgCl_2_, 0.1% Igepal CA-630 and 5% glycerol), and complexes were eluted by incubation in 1 CV of wash buffer 1 supplemented with 10% (v/v) of TEV protease (1 mg ml^−1^), for 3 h at room temperature. A second-step purification was performed by incubation FLAG eluate with 5% total volume of Pierce high-capacity streptavidin agarose for 3 h at 8 °C with shaking. Beads were washed three times with 10 CV of the wash buffer 2 (20 mM K-HEPES pH 7.9, 500 mM KCl, 2 mM MgCl_2_). Samples were eluted from the resin by several incubations with 0.5 CV of wash buffer 2 supplemented with 10 mM biotin for 15 min on ice. The eluate was loaded onto a 4 ml 10–30% glycerol gradient containing 20 mM K-HEPES pH 7.9, 500 mM KCl, 2 mM MgCl_2_, 0.1% Igepal CA-630 and 0–0.1% glutaraldehyde^33^ and centrifuged for 16 h at 35,000 rpm at 4 °C (Beckman Coulter Ultracentrifuge Optima L-90K). The peak fraction of the glycerol gradient was analyzed by negative staining EM, and the fractions containing most homogeneous particles were dialyzed against a buffer containing 20 mM K-HEPES pH 7.9, 150 mM KCl and 2 mM MgCl_2_ and used directly for grid preparation without further manipulations.

### Cryo-EM data collection and processing

The sample was applied to 300 mesh Quantifoil R 1.2/1.3 grids covered with 3 nm continuous carbon, which had been glow-discharged for 30 s at 15 mA at 0.4 mbar using the Pelco EasiGlow. The grids were plunge frozen in liquid ethane after applying 2 µl at 4 °C, 100 % humidity and blotting for 2 s at blot force −5 in a Vitrobot Mark IV. The grids were screened on a Glacios 200 kV microscope equipped with a Falcon III detector and transferred to a Titan Krios microscope operating at 300 kV equipped with a Gatan Energy filter^34^. A total of 8,506 micrographs were recorded using SerialEM^35^ and a K2 direct electron detector at a magnification of 130,000×, a defocus between −1.5 and −3.5 μm with a dose rate of 4.6 e^−^ per pixel per second and inserted energy slit at 20 eV, as well as the 70 μm objective aperture. The total dose was 40.5 e^−^ Å^−^^2^, accumulated in 40 frames at a final pixel size of 1.045 Å. All image processing was done using cryoSPARC v3.3 (ref. ^36^). For preprocessing, we used patch motion correction and determined the contrast transfer function (CTF) parameters using patch CTF estimation. Using the blob picker functionality, 503,581 particles were picked and extracted in a 504-pixel box. After binning two times, the particles were subjected to two-dimensional classification to create templates for template picking, which resulted in 490,503 picked particles. These particles were subjected to two-dimensional classification, ab initio reconstruction, followed by three-dimensional structure heterogeneous refinement until a homogeneous subpopulation of 76,918 particles was identified. Nonuniform refinement resulted in a final 3.1 Å resolution map based on the 0.143 Fourier Shell Correlation (FSC) criterion^37,38^. The obtained map was sharpened by applying a B-factor of −55 Å^2^.

### Model building and structural analysis

Atomic coordinates of the U5 snRNP components extracted from the structure of the human tri-snRNP^21^ (PDB ID: 6qw6) were used as templates for modeling. Individual chains were fitted into the cryo-EM density as rigid bodies using UCSF Chimera^39^, the components with well-resolved density were manually adjusted and rebuilt in Coot v0.9.8.5 (ref. ^40^). Other components with poorly resolved densities (that is, BRR2, PRP8^RNaseH^, PRP8^Jab1/MPN^, DDX23, Sm ring, 40K) were docked into the map as rigid bodies and left in their original form. CD2BP2 binding sites were initially identified by an exhaustive in silico AlphaFold2-based search^41,42^ for all possible interactions with other U5 snRNP components, using a previously described approach^43^.

Atomic models were initially refined with Refmac Servalcat v5.8.0267 (ref. ^44^) with secondary structure restraints generated with ProSMART^45^ via the CCP-EM software suite^46^. Final models were refined in real space in Phenix^47^ and validated in Molprobity^48^. Structural representations for figures were prepared with Pymol (Schrödinger) and ChimeraX^49^.

### Cross-linking and mass spectrometry analysis

CD2BP2 complex at 3 mg ml^−1^ was incubated with 0.25 mM or 1 mM BS3 for 30 min at 30 °C with shaking at 600 rpm (ThermoMixer, Eppendorf), and the cross-linking reaction was quenched by the addition of Tris-Cl pH 7.5 to the final concentration of 50 mM and incubated for 10 min at 35 °C at 600 rpm. Then, samples were mixed with 0.05 (v/v) of RapiGest and, after the addition of 10 mM DTT, incubated at 50 °C for 30 min, with shaking at 600 rpm. Subsequently, 2-chloroacetamide was added to 50 mM final concentration, and samples were incubated at 25 °C for 30 min at 600 rpm, protected from direct light. Proteins were digested with 1:50 (m/m ratio) of trypsin and 1:100 of LysC for 16 h at 37 °C. Digestion was stopped by adding 0.5% (v/v) of trifluoroacetic acid. Further analysis was performed by EMBL Proteomics Core Facility in Heidelberg.

Digested peptides were concentrated and desalted using an OASIS HLB µElution Plate (Waters), according to manufacturer instructions. Crosslinked peptides were enriched using size exclusion chromatography^50^. In brief, desalted peptides were reconstituted with size exclusion chromatograph buffer (30% (v/v) acetonitrile (ACN) in 0.1% (v/v) trifluoroacetic acid (TFA)) and fractionated using a Superdex Peptide PC 3.2/30 column (GE) on a 1200 Infinity high-performance liquid chromatography system (Agilent) at a flow rate of 0.05 ml min^−1^. Fractions eluting between 50–70 μl were evaporated to dryness and reconstituted in 30 μl 4% (v/v) ACN in 1% (v/v) FA.

Collected fractions were analyzed by liquid chromatography‐coupled tandem mass spectrometry using an UltiMate 3000 RSLC nano liquid chromatography system (Dionex) fitted with a trapping cartridge (µ-Precolumn C18 PepMap 100, 5 µm, 300 µm × 5 mm, 100 Å) and an analytical column (nanoEase M/Z HSS T3 column 75 µm × 250 mm C18, 1.8 µm, 100 Å, Waters). Trapping was carried out with a constant flow of trapping solvent (0.05% trifluoroacetic acid in water) at 30 µl min^−1^ onto the trapping column for 6 min. Subsequently, peptides were eluted and separated on the analytical column using a gradient composed of solvent A (3% dimethyl sulfoxide and 0.1% formic acid in water) and solvent B (3% dimethyl sulfoxide and 0.1% formic acid in acetonitrile) with a constant flow of 0.3 µl min^−1^. The outlet of the analytical column was coupled directly to an Orbitrap Fusion Lumos (Thermo Scientific) mass spectrometer using the nanoFlex source.

The peptides were introduced into the Orbitrap Fusion Lumos via a Pico-Tip Emitter 360 µm × 20 µm; 10 µm tip (CoAnn Technologies) and an applied spray voltage of 2.1 kV, and the instrument was operated in positive mode. The capillary temperature was set at 275 °C. Only charge states of 4–8 were included. The dynamic exclusion was set to 30 s and the intensity threshold was 5 × 10^4^. Full mass scans were acquired for a mass range 350–1,700 *m*/*z* in profile mode in the orbitrap with resolution of 120,000. The AGC target was set to standard and the injection time mode was set to auto. The instrument was operated in data-dependent acquisition mode with a cycle time of 3 s between master scans and tandem mass spectrometry (MS/MS) scans were acquired in the Orbitrap with a resolution of 30,000, with a fill time of up to 100 ms and a limitation of 2 × 10^5^ ions (AGC target). A normalized collision energy of 32 was applied. MS2 data were acquired in profile mode.

All data were analyzed using the cross-linking module in Mass Spec Studio v2.4.0.3524 (www.msstudio.ca, ref. ^51^). Parameters were set as follows: trypsin (K/R only), charge states 3–8, peptide length 7–50, percent *E*-value threshold of 50, mass spectrometry (MS) mass tolerance of 10 ppm, tandem mass spectrometry mass tolerance of 10 and elution width of 0.5 min. BS3 cross-links residue pairs were constrained to KSTY on one end and one of KSTY on the other. Identifications were manually validated, and cross-links with an *E*-value corresponding to <0.05% false discovery rate (FDR) were rejected. The data export from the Studio was filtered to retain only cross-links with a unique pair of peptide sequences and a unique set of potential residue sites.

Structural and functional analysis of the XL-MS data were performed with XiView^52^.

### Reporting summary

Further information on research design is available in the Nature Portfolio Reporting Summary linked to this article.

## Online content

Any methods, additional references, Nature Portfolio reporting summaries, Source data, Extended data, Supplementary Information, acknowledgements, peer review information; details of author contributions and competing interests; and statements of data and code availability are available at 10.1038/s41594-024-01250-5.

### Supplementary information

Reporting Summary

Peer Review File

### Source data

Source Data Fig. 1 Source data for the quantitative proteomics volcano plot are shown in Fig. 1a.

Source Data Extended Data Fig. 1 Unprocessed western blots used in Extended Data Fig. 1.

Source Data Extended Data Fig. 2 Unprocessed sodium dodecyl sulfate polyacrylamide gel electrophoresis and western blots used in Extended Data Fig. 2.

Source Data Extended Data Fig. 8 Unprocessed western blots used in Extended Data Fig. 8f.

Source Data Extended Data Fig. 9 List of peptides and peptides pair identified in the XL-MS experiment of the 20S U5 snRNP sample using 0.25 mM and 1 mM BS3.

## Figures and Tables

**Extended Data Table 1:**
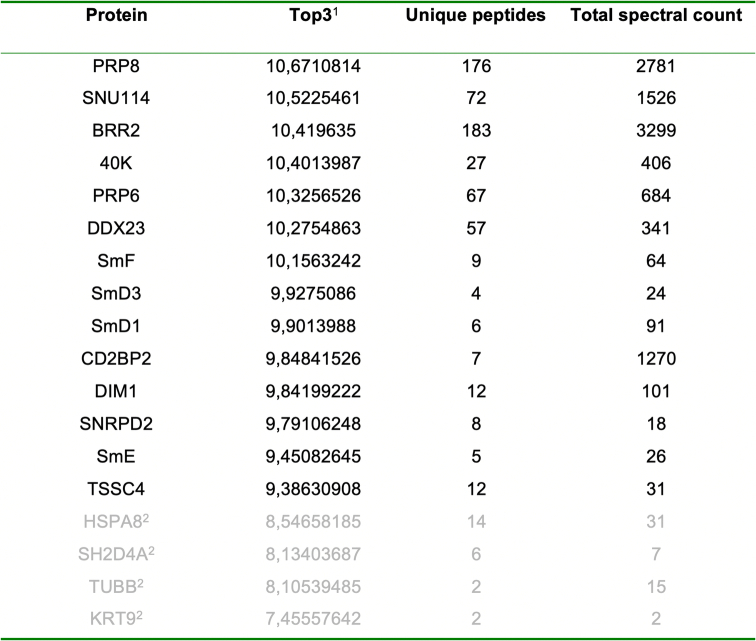
Solution mass spectrometry analysis of the purified 20 S U5 snRNP

Proteins in the table were sorted based on their Top3 values in a descending order. ^1^The Top3 value is the average log10 MS1 intensity of the three most abundant peptides for each protein and serves as an estimator for the average abundance. ^2^These proteins are typical contaminants present in the preparation.

## Data Availability

Structural data have been deposited in PDB and EMDB under the following accession codes: PDB 8Q91 and EMD-18267 for the 20S U5 snRNP core structure and PDB 8RC0 and EMD-19041 for the complete model of the 20S U5 snRNP. Other atomic coordinates used in this study for the comparisons purposes are available from the PDB under the following accession codes: 6QW6 for the U4/U6.U5 tri-snRNP, 6FF4 for the Bact complex; 6ID0 for the human ILS complex and 4I43 for AAR2–PRP8 complex. Quantitative proteomics and XL-MS data are provided as source data together with this manuscript. Other data and materials created within this study will be made available upon request. Source data are provided with this paper.
