# Supplementary material for: Structure of the human 20S U5 snRNP
Source: Nat Struct Mol Biol. 2024 Mar 11;31(5):752–6. doi: 10.1038/s41594-024-01250-5 (PMC11102862; doi:10.1038/s41594-024-01250-5)
Supplement: Supplementary file 1 — Reporting Summary [file 41594_2024_1250_MOESM1_ESM.pdf]

nature portfolio

Corresponding author(s): Wojciech Galej

Last updated by author(s): 20/01/2024

Reporting Summary

Nature Portfolio wishes to improve the reproducibility of the work that we publish. This form provides structure for consistency and transparency in reporting. For further information on Nature Portfolio policies, see our Editorial Policies and the Editorial Policy Checklist.

Statistics

For all statistical analyses, confirm that the following items are present in the figure legend, table legend, main text, or Methods section.

n/a	Confirmed	
☒	☐	The exact sample size (*n*) for each experimental group/condition, given as a discrete number and unit of measurement
☒	☐	A statement on whether measurements were taken from distinct samples or whether the same sample was measured repeatedly
☐	☒	The statistical test(s) used AND whether they are one- or two-sided<br>*Only common tests should be described solely by name; describe more complex techniques in the Methods section.*
☒	☐	A description of all covariates tested
☒	☐	A description of any assumptions or corrections, such as tests of normality and adjustment for multiple comparisons
☒	☐	A full description of the statistical parameters including central tendency (e.g. means) or other basic estimates (e.g. regression coefficient) AND variation (e.g. standard deviation) or associated estimates of uncertainty (e.g. confidence intervals)
☐	☒	For null hypothesis testing, the test statistic (e.g. *F*, *t*, *r*) with confidence intervals, effect sizes, degrees of freedom and *P* value noted<br>*Give P values as exact values whenever suitable.*
☒	☐	For Bayesian analysis, information on the choice of priors and Markov chain Monte Carlo settings
☒	☐	For hierarchical and complex designs, identification of the appropriate level for tests and full reporting of outcomes
☒	☐	Estimates of effect sizes (e.g. Cohen's *d*, Pearson's *r*), indicating how they were calculated

Our web collection on statistics for biologists contains articles on many of the points above.

Software and code

Policy information about availability of computer code

Data collection	SerialEM, Mass Spec Studio v2.4.0.3524
Data analysis	CryoSPARC v3.3; UCSF Chimera 1.5; Coot v0.9.8.5; Refmac Servalcat v5.8.0267; Pymol 2.5.5; PHENIX 1.20.1_4487; XiView; cryoSPARC v3.3

For manuscripts utilizing custom algorithms or software that are central to the research but not yet described in published literature, software must be made available to editors and reviewers. We strongly encourage code deposition in a community repository (e.g. GitHub). See the Nature Portfolio guidelines for submitting code & software for further information.

Data

Policy information about availability of data

All manuscripts must include a data availability statement. This statement should provide the following information, where applicable:

- Accession codes, unique identifiers, or web links for publicly available datasets
- A description of any restrictions on data availability
- For clinical datasets or third party data, please ensure that the statement adheres to our policy

Structural data has been deposited in PDB and EMDB under the following accession codes: PDB 8Q91 and EMD-18267 for the 20S U5 snRNP core structure and PDB 8RC0 and EMD-19041 for the complete model of the 20S U5 snRNP. Other atomic coordinates used in this study for the comparisons purposes are available from the PDB under the following accession codes: 6QW6 for the U4/U6.U5 tri-snRNP, 6FF4 for the Bact complex; 6ID0 for the human ILS complex and 4I43 for Aar2-Prp8 complex. Other data and materials created within this study will be made available upon request.

Research involving human participants, their data, or biological material

Policy information about studies with human participants or human data. See also policy information about sex, gender (identity/presentation), and sexual orientation and race, ethnicity and racism.

Reporting on sex and gender	N/A
Reporting on race, ethnicity, or other socially relevant groupings	N/A
Population characteristics	N/A
Recruitment	N/A
Ethics oversight	N/A

Note that full information on the approval of the study protocol must also be provided in the manuscript.

Field-specific reporting

Please select the one below that is the best fit for your research. If you are not sure, read the appropriate sections before making your selection.

☒ Life sciences ☐ Behavioural & social sciences ☐ Ecological, evolutionary & environmental sciences

For a reference copy of the document with all sections, see nature.com/documents/nr-reporting-summary-flat.pdf

Life sciences study design

All studies must disclose on these points even when the disclosure is negative.

Sample size	cryo-EM data sample size was determined by the data processing outcome yielding a desired resolution.
Data exclusions	Some of the cryo-EM data was excluded during classification procedures following common practices in this field.
Replication	Quantitative proteomics experiments were performed in three biological replicates.
Randomization	Two half-datasets used to calculate gold-standard FSC curves were assigned randomly.
Blinding	Investigators were blinded with respect to half-datasets assignments.

Reporting for specific materials, systems and methods

We require information from authors about some types of materials, experimental systems and methods used in many studies. Here, indicate whether each material, system or method listed is relevant to your study. If you are not sure if a list item applies to your research, read the appropriate section before selecting a response.

Materials & experimental systems

n/a	Involved in the study	
☐	☒	Antibodies
☐	☒	Eukaryotic cell lines
☒	☐	Palaeontology and archaeology
☒	☐	Animals and other organisms
☒	☐	Clinical data
☒	☐	Dual use research of concern
☒	☐	Plants

Methods

n/a	Involved in the study	
☒	☐	ChIP-seq
☒	☐	Flow cytometry
☒	☐	MRI-based neuroimaging

Antibodies

Antibodies used	Anti-2,2,7-Trimethylguanosine Mouse antibodies, K121, Agarose Conjugate, Merck NA02A<br>CD2BP2 (Sigma, HPA061309), 1:5000<br>DIM1 (Proteintech, 27646-1-AP), 1: 3000<br>PRP6 (Invitrogen, PA5-61428), 1: 2000

	SNU114/EFTUD2 (Invitrogen, PA5-96559) 1:5000<br>goat anti-rabbit IgG H&L, HRP (Abcam, ab205718), 1:5000<br>Anti-FLAG M2-Peroxidase (Sigma, A8592), 1: 5000<br>anti-GAPDH (Invitrogen MA515738HRP), 1:5000<br>anti-HA-Tag F-7 (SantaCruz, sc-7392), 1:5000
Validation	validation performed by manufacturers

Eukaryotic cell lines

Policy information about cell lines and Sex and Gender in Research

Cell line source(s)	HEK293T (ATCC),Freestyle 293 cells (ThermoFisher)
Authentication	cell lines were not authenticated
Mycoplasma contamination	not tested
Commonly misidentified lines<br>(See ICLAC register)	*Name any commonly misidentified cell lines used in the study and provide a rationale for their use.*

Plants

Seed stocks	*Report on the source of all seed stocks or other plant material used. If applicable, state the seed stock centre and catalogue number. If plant specimens were collected from the field, describe the collection location, date and sampling procedures.*
Novel plant genotypes	*Describe the methods by which all novel plant genotypes were produced. This includes those generated by transgenic approaches, gene editing, chemical/radiation-based mutagenesis and hybridization. For transgenic lines, describe the transformation method, the number of independent lines analyzed and the generation upon which experiments were performed. For gene-edited lines, describe the editor used, the endogenous sequence targeted for editing, the targeting guide RNA sequence (if applicable) and how the editor was applied.*
Authentication	*Describe any authentication procedures for each seed stock used or novel genotype generated. Describe any experiments used to assess the effect of a mutation and, where applicable, how potential secondary effects (e.g. second site T-DNA insertions, mosiacism, off-target gene editing) were examined.*
