# Supplementary figures and images for: Structure of the human 20S U5 snRNP
Source: Nat Struct Mol Biol. 2024 Mar 11;31(5):752–6. doi: 10.1038/s41594-024-01250-5 (PMC11102862; doi:10.1038/s41594-024-01250-5)

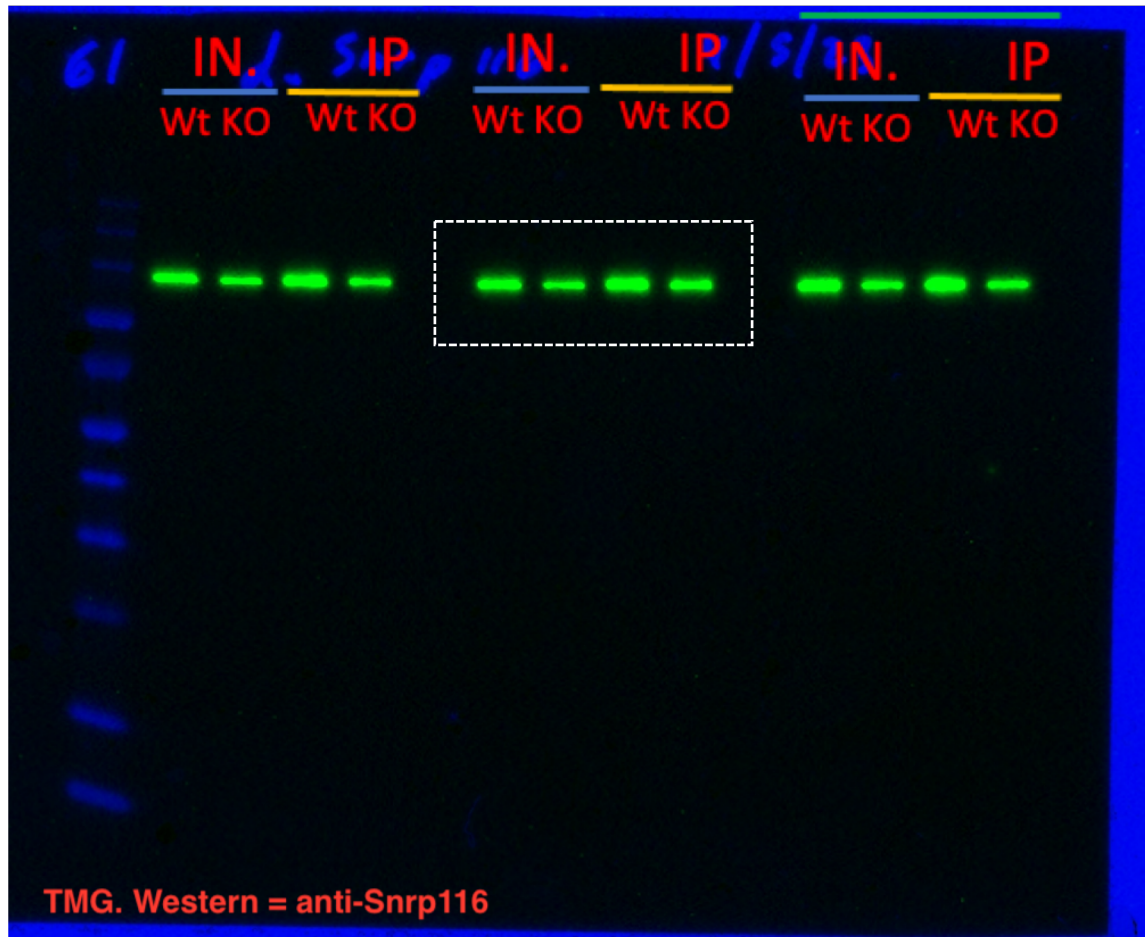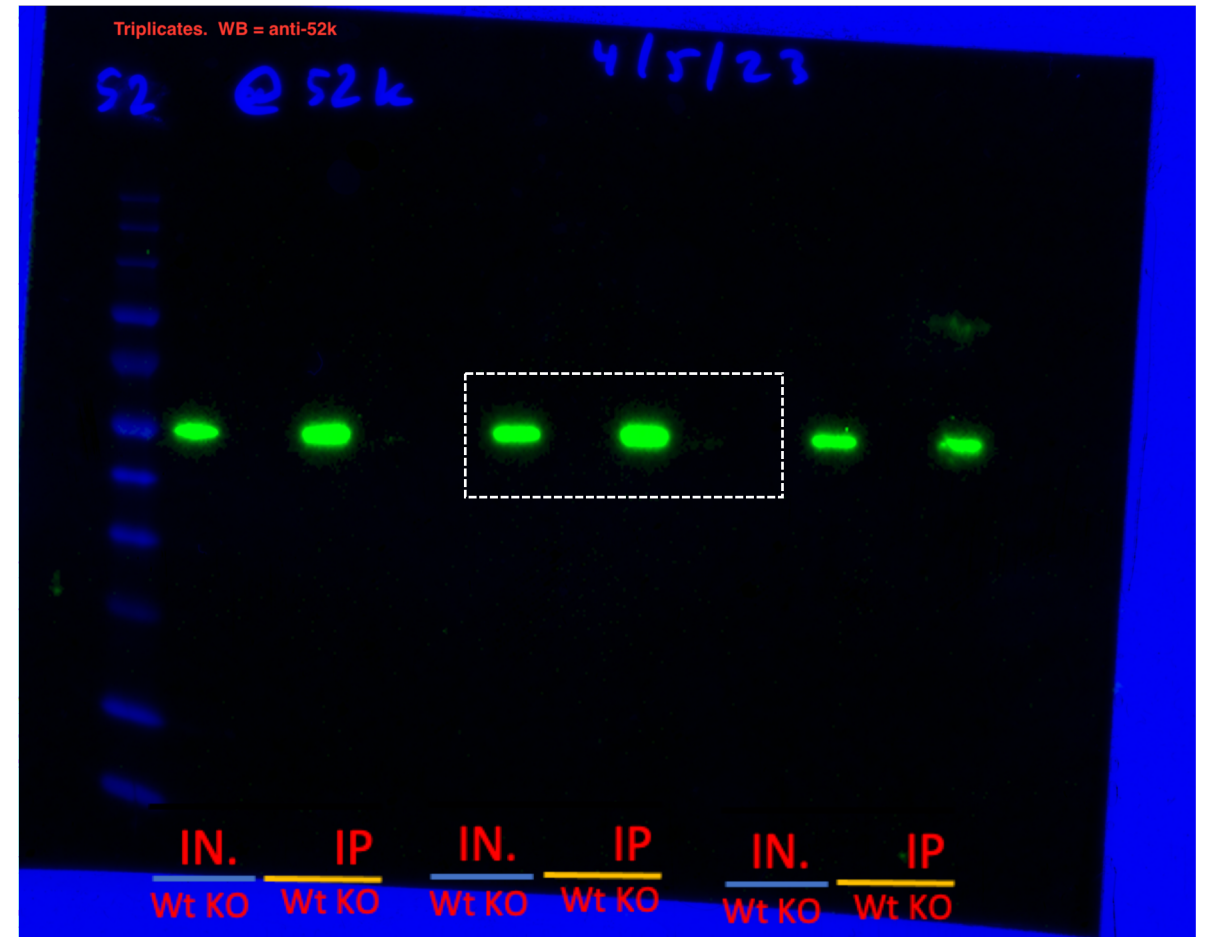

WT

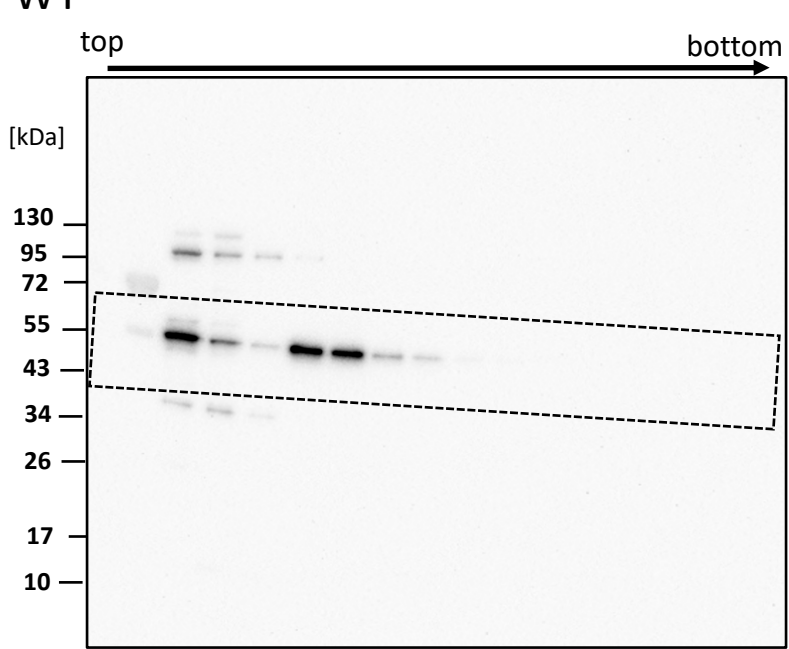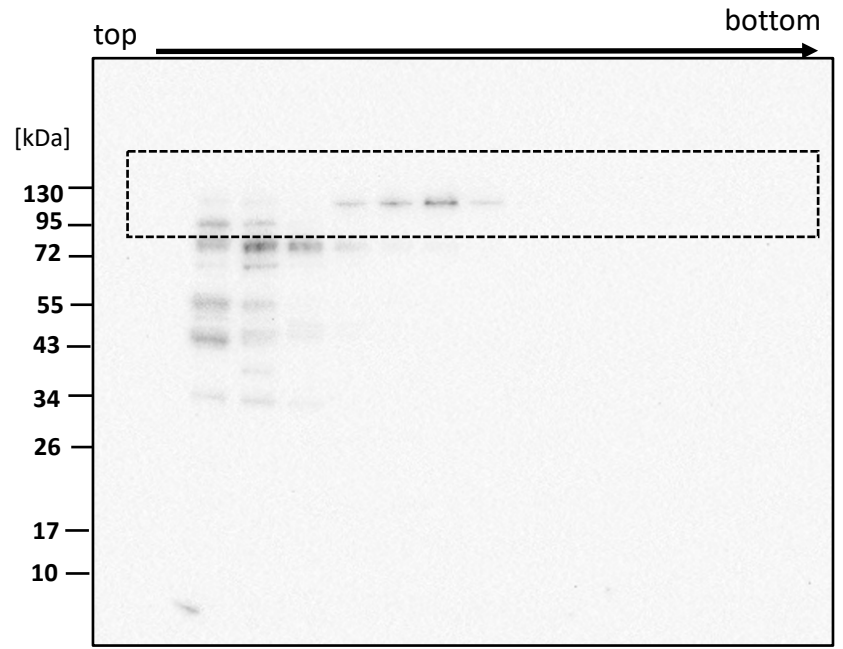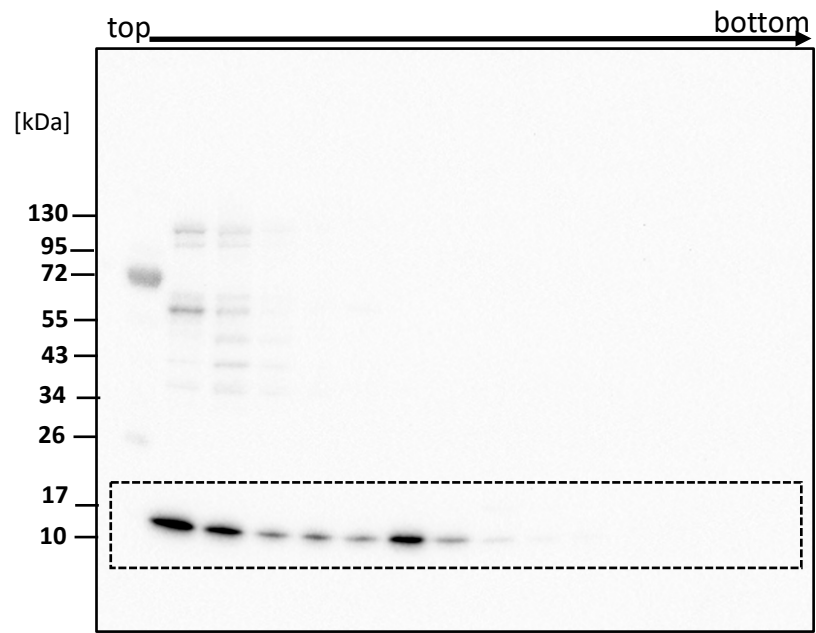

CD2BP2<sup>KO</sup>

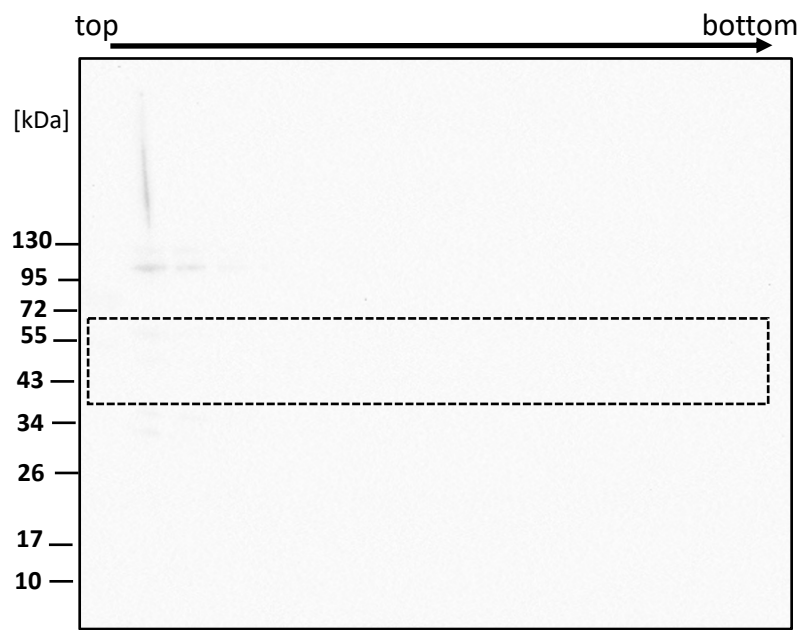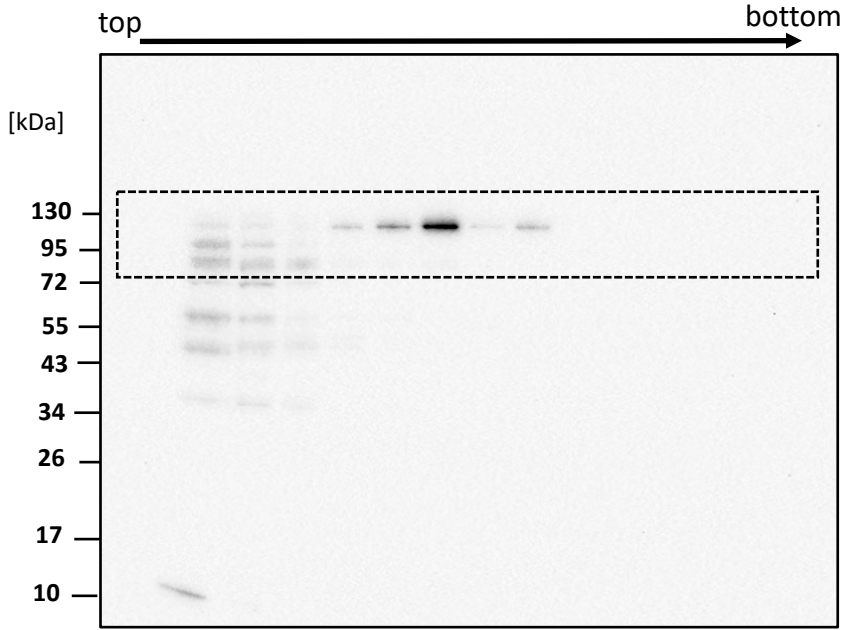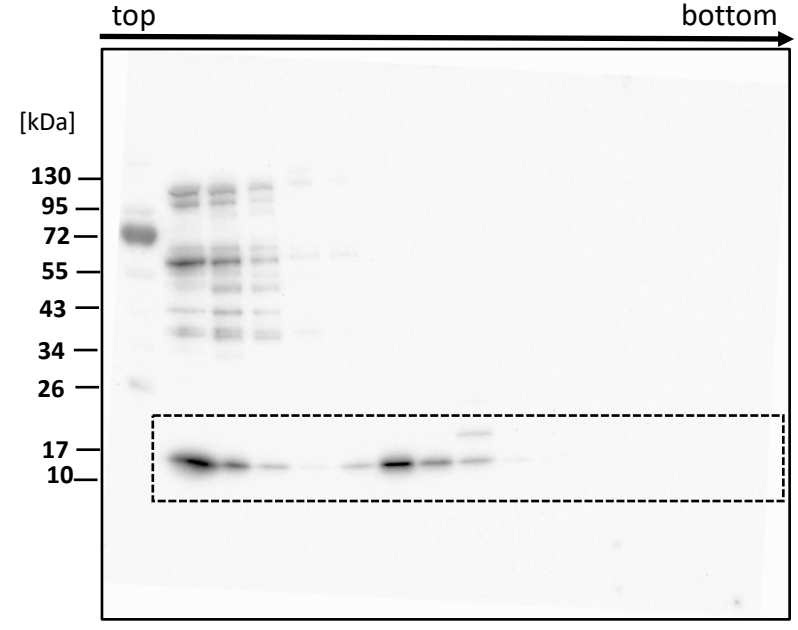

Supplement: Supplementary file 4 — Unprocessed western blots used in Extended Data Fig. 1. [file 41594_2024_1250_MOESM4_ESM.pdf]

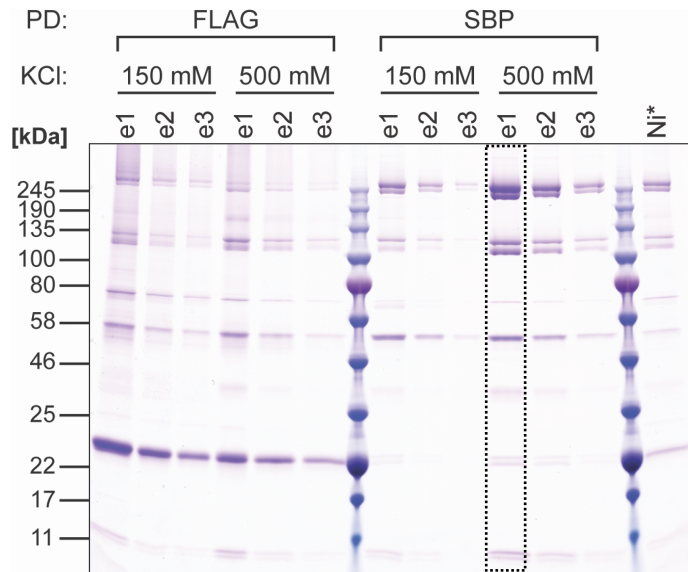

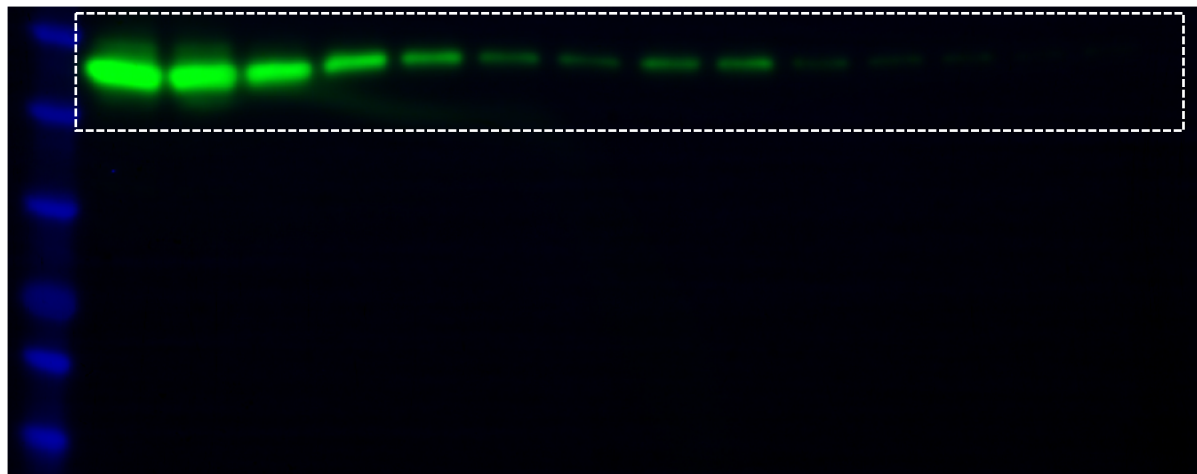

Supplement: Supplementary file 5 — Unprocessed sodium dodecyl sulfate polyacrylamide gel electrophoresis and western blots used in Extended Data Fig. 2. [file 41594_2024_1250_MOESM5_ESM.pdf]

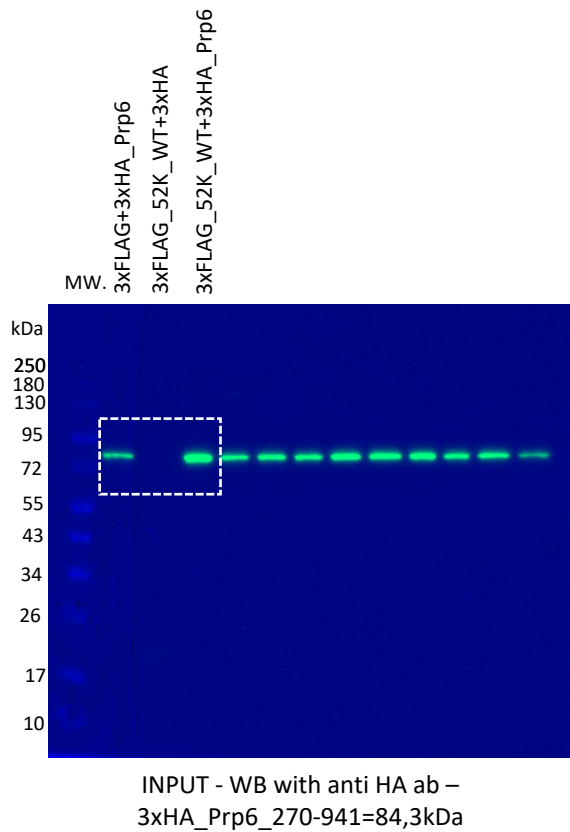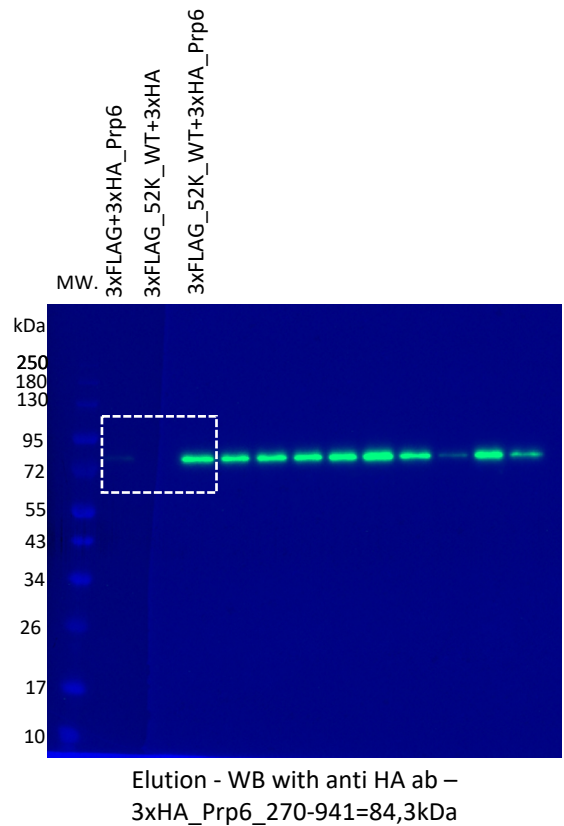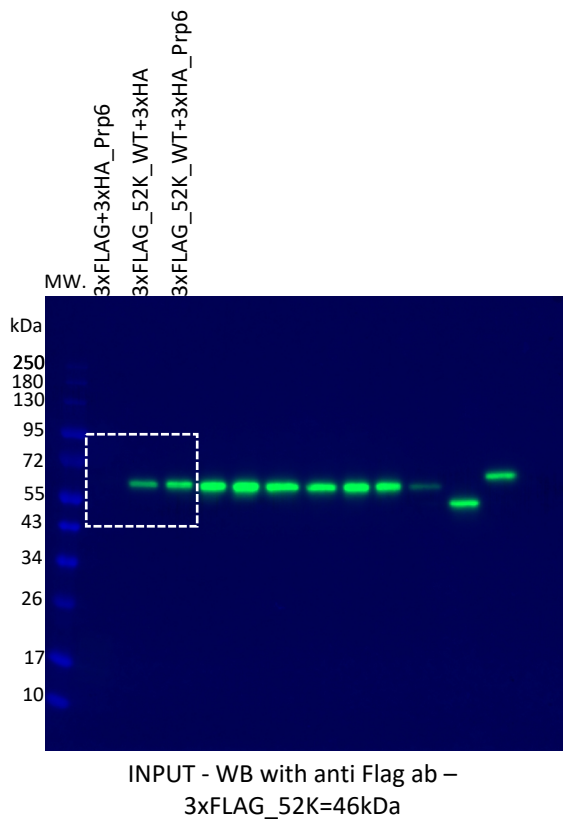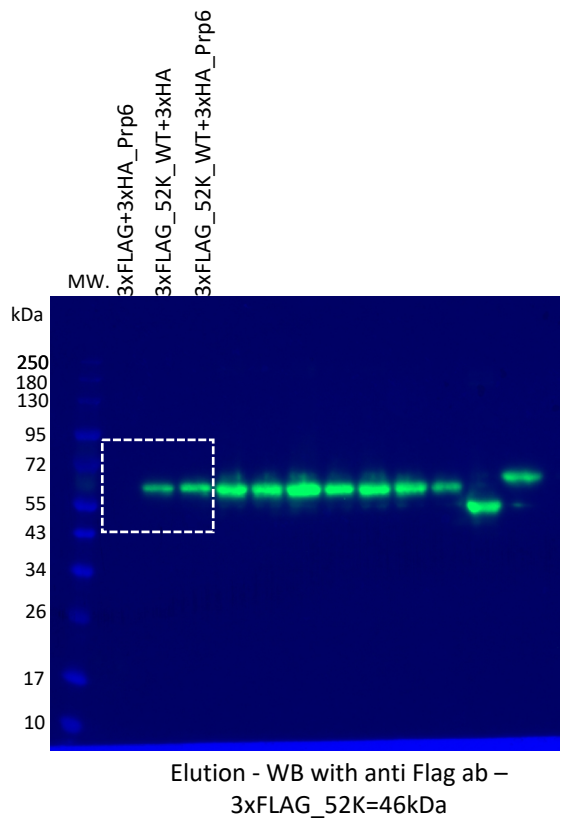

Supplement: Supplementary file 6 — Unprocessed western blots used in Extended Data Fig. 8f. [file 41594_2024_1250_MOESM6_ESM.pdf]
